# Varying perceptions of the role of “nurse as teacher” for medical trainees: A qualitative study

**DOI:** 10.1007/s40037-020-00632-x

**Published:** 2020-12-03

**Authors:** Asif Doja, Carolina Lavin Venegas, Chantalle Clarkin, Katherine Scowcroft, Gerry Ashton, Laura Hopkins, M. Dylan Bould, Hilary Writer, Glenn Posner

**Affiliations:** 1 Faculty of Medicine, University of Ottawa, Ottawa, Ontario Canada; 2 Children’s Hospital of Eastern Ontario, Ottawa, Ontario Canada; 3 Children’s Hospital of Eastern Ontario Research Institute, Ottawa, Ontario Canada; 4 Centre for Addiction and Mental Health, Toronto, Ontario Canada; 5 The Ottawa Hospital, Ottawa, Ontario Canada; 6 Division of Oncology, University of Saskatchewan, Saskatoon, Saskatchewan Canada

**Keywords:** Nursing, Informal curriculum, Teaching, Undergraduate medical education, Qualitative methodology

## Abstract

**Introduction:**

The informal curriculum—an essential complement to the formal curriculum—is delivered to medical trainees through learning outside the classroom. We sought to explore nurse-mediated aspects of trainee education in the informal curriculum in obstetrics and gynecology (OBGYN), as well as nursing perceptions of their role in medical trainee education.

**Methods:**

Naturalistic, non-participant observations (40 h) were performed on a tertiary care birthing unit (BU) to document teaching and learning interactions. Insights gleaned from observations informed subsequent semi-structured interviews with BU nurses (*n* = 10) and focus group discussions with third-year medical students who had completed an OBGYN rotation (*n* = 10). Thematic analysis was conducted across data sets.

**Results:**

Conceptions of nurse-mediated education differed considerably between nurses and trainees. Nurses were widely acknowledged as gatekeepers and patient advocates by both groups, although this role was sometimes perceived by trainees as impacting on learning. Interest and engagement were noted as mediators of teaching, with enhanced access to educational opportunities reported by trainees who modelled openness and enthusiasm for learning. Nurse-driven education was frequently tailored to the learner’s level, with nurses feeling well positioned to share procedural knowledge or *hard skills, soft skills* (i.e. bedside manners), and clinical insights gained from bedside practice.

**Discussion:**

Nurses are instrumental in the education of medical trainees; however, divergence was noted in how this role is enacted in practice. Given the valuable teaching resource BU nurses present, more emphasis should be placed on interprofessional co-learning and the actualization of this role within the informal curriculum.

## Introduction

The education of medical trainees is not limited to classroom or labs; it occurs on wards, at the bedside, and in clinical environments where students function as members of larger interprofessional teams. The formal curriculum involves the planned teaching of competencies in relation to the explicitly stated goals and objectives of undergraduate or postgraduate medical programs, including learning outside the classroom. The informal curriculum, an essential complement, refers to unscripted, ad hoc, interpersonal forms of learning occurring via encounters with faculty or other health professionals [1]. The hidden curriculum, often unexplored, consists of what is taught to trainees without intending or being aware it is taught. All three forms of education can influence, to varying degrees, a trainee’s learning and how they conceptualize healthcare culture, their roles, and future performance as physicians.

The informal curriculum of learning outside the classroom (i.e. work settings) has been shown to make distinct contributions to a trainee’s medical education as well as to their continuing educational development [2]. However, learning in the informal curriculum needs to be considered differently from “classroom learning” [2].

For example, students will only learn in clinical environments if they are sufficiently engaged [3]. The close personal interaction in the informal curriculum is a key component of this engagement. Highly invitational opportunities have the potential to advance learning, whereas being denied access to certain activities may hinder learning [3]. Nurses have the potential to play an important role in the informal curriculum, but scarce literature exists on the direct teaching that nurses deliver to medical students. Anecdotally, many physicians report significant learning from nurses [4], and observational research provides evidence that nurses contribute substantially to the interprofessional education of residents in clinical settings [5]. However, some research suggests a lack of clarity between nurses and medical students regarding educational roles and learning objectives. Capstick and Harley [6] surveyed 87 birthing unit (BU) nurses to examine their attitudes towards teaching medical students. They concluded that their institutional program lacked clear objectives—endorsed by everyone (including nurses) involved in teaching—for medical students. For example, nurses in this study had different perceptions of the clinical experiences students should have during an obstetrics and gynecology (OBGYN) rotation [6], with 43% of nurses deeming it inappropriate for medical students to perform outpatient assessments and pelvic exams during labour, and 9% believing medical students should not perform supervised deliveries [6].

Another study surveyed medical students and nurses to evaluate the quality of their interactions and found that medical students rated their interactions with nurses lower than interactions with residents or attendings—and vice versa [7]. Unclear expectations and conflicting views regarding the “nurse as teacher role”, coupled with perceptions of poor-quality interactions between nurses and medical students may disrupt and impair interprofessional education and collaboration. A recent systematic review of the perceptions of residents, medical students, and nursing students about interprofessional education revealed that low-quality interactions negatively impacted collaboration, as did the lack of familiarity with each other’s roles [8].

Thus, while it is acknowledged that nurses routinely participate in the education of medical trainees, little is described about the enactment of this role and the complex interprofessional relationships that take unfold within the informal curriculum. As such, we sought to examine the ways in which medical trainees learn from nurses on a BU and explore how nurses perceive their role in the education of medical trainees.

## Methods

This exploratory, qualitative study examined nurse-mediated education of third-year medical trainees during their OBGYN rotation. Data collection methods documented examples of teaching and learning, as well as trainee and nurse perceptions, through: (1) naturalistic observations of the interactions between medical trainees and nurses on the BU, (2) focus group discussion with third-year medical students who had recently completed an OBGYN rotation, and (3) one-on-one-interviews with nursing staff. Observation data was used to inform semi-structured interview and focus group questions. The interviews and focus group discussions allowed for the clarification and elaboration of process, practice issues, and meanings, including elements that enriched or inhibited learning in the BU setting.

As a setting for study, the BU is rich with educational opportunity. Firstly, it is a unique environment which requires skills that are not readily transferable from other specialties. As such, BU nurses develop specialized knowledge that they can impart on medical trainees. Secondly, the BU includes both medical and surgical aspects. The operating room (OR) environment is another similarly specialized area with its own code of conduct [9]. BU nurses function as OR nurses when their patients require a caesarian section; thus, the same nurses often engage with trainees in more than one clinical environment.

### Study site and participant recruitment

Observations were conducted on a BU at a high-volume (~3000 deliveries per year), acute-care, tertiary teaching hospital with a level‑3 nursery. Sixty third-year medical students rotate through the BU annually during their OBGYN clerkship rotation.

We used a convenience approach to recruit participants during the observation period. Informed consent was obtained from attending staff physicians and members of the multidisciplinary healthcare team prior to the observations. Participation was voluntary and notes were not collected on those who declined observation.

Following the collection and preliminary analysis of field notes, we used a purposive-snowball approach (i.e. identify and seek volunteers) to recruit third-year medical students for a one-hour focus group session. Focus group methods were selected to encourage conversation and dynamic interactions between participants, and to collect a range of experiences and perspectives [10]. Recruitment was facilitated by the OBGYN undergraduate program coordinator, who circulated an email invitation to third-year medical students who had recently completed an OBGYN rotation.

One-on-one interviews were conducted with a purposive sample of nurses who participated in the observations and had documented interactions with medical trainees. These nurses were approached for participation by the BU nurse educator (GA). Interviews were conducted in-person on the unit, (duration: 15–20 min). Informed consent was obtained prior to the focus groups and interviews.

This study was approved by the Children’s Hospital of Eastern Ontario Research Ethics Board (Protocol no. 15/153X) and the Ottawa Hospital Research Ethics Board (Protocol no. 20140669-01H).

### Data collection

Observations were conducted by a research assistant over a period of 10 consecutive business days (total: 40 h). Field notes were collected and were summarized following each session to capture reflective insights. The observer was instructed to document trainee-nurses interactions focusing on interprofessional dynamics and educational opportunities, noting details regarding location, timing, presence of other providers, presence of patients and caregivers, and verbal and non-verbal reactions. All observation notes were collected on paper, then transcribed during summation, and finally imported into *NVivo11* (QSR International, Melbourne, Australia) for data management.

Semi-structured focus group sessions were moderated by a trained research assistant, with a note-taker documenting dynamics such as dominant speakers, group reactions, and non-verbal cues. Each session began with an introductory definition of the informal curriculum. Questions explored aspects of educational interactions in order to better understand experiences and perceptions of teaching and learning, the type and content of educational interactions that occurred, and factors that encouraged or inhibited nurse-mediated learning in the BU. Sessions were audio-recorded and the research assistant and note-taker discussed impressions following each session.

Individual, semi-structured interviews were conducted by a trained research assistant. The interview guide was informed by preliminary insights from the observation period, and questions were designed to explore how nurses conceptualized their role as medical educators, perceived trainee learning needs and expectations, and what content they felt the trainees should know about the BU. Interviews were audio-recorded for verbatim transcription.

### Data analysis

Data collection and analysis followed an iterative process, with recurrent review of data as understandings deepened [11]. Two researchers trained in qualitative methods (CC and CLV) initially independently reviewed field notes and summaries, interview transcripts, and focus group transcripts. During this process, data were reviewed line-by-line to capture key concepts. The researchers then met to discuss impressions of the data, compare and contrast interpretations within and across data sets, and develop a shared coding scheme featuring themes and subthemes. They also discussed their underlying assumptions and biases as an awareness raising activity. Data were then coded systematically according to the scheme while allowing for emergence of new previously unseen concepts. The researchers (AD, CLV, CC) discussed each transcript to review the coding, resolve conflicting interpretations, revise concepts, and update the coding scheme. Disagreements were discussed among the whole team until consensus was achieved. When coding was complete, the team met to review the findings, clarify interpretations, and refine concepts. They also discussed whether any concepts/themes were missed, if additional lines of inquiry should be explored, and confirmed that informational redundancy was met.

### Rigor and trustworthiness of data

*Credibility, transferability, dependability*, and *confirmability *strategies were enacted to demonstrate trustworthiness of the data [12]. Peer review, to enact *credibility*, was achieved by presenting the study at a local medical education conference. To enact *transferability*, contextual details and rich description of data were provided, including the context of the study, the participants, the data collection and processes of analysis. *Dependability *of findings was enacted through investigator triangulation, with researchers from both nursing and medical backgrounds with different levels of training performing analytic decision-making and interpretations. Data was collected from multiple sources and through varied collection methods. To ensure *confirmability* of the study, coding decisions and changes to the coding manual were continuously audited.

## Results

Observations were recorded on the BU, in a variety of sub-areas including the triage area, patients’ rooms, nurses’ station, and hallway. Interactions were observed between nurses, medical students, clerks, residents, and physicians. Three focus groups were conducted with a total of 10 third-year medical students (3 female, 7 male). Interviews were conducted with ten nurses (female) working at the same BU.

Four major themes were identified from the analysis, highlighting differing conceptualizations of nurses’ educational role: (1) *nurses’ role as gatekeepers and patient advocates,* (2) *engagement as a facilitator of teaching,* (3) *tailoring teaching to the learner,* and (4) *differences in the understanding of teaching offered by nurses in the informal curriculum.*

### Nurses’ role as gatekeepers and patient advocates

When discussing their roles, nurses used language that highlighted their roles as gatekeepers or protective patient advocates, including: *“protective of our patients”, *“*we’re defenses”, “our role is to keep patients safe”. *When describing this concept further, they mentioned stopping trainees from entering patients’ room if they felt the timing or circumstances were not appropriate, and recalled *“slowing down”* overconfident and/or inexperienced trainees to encourage them to think critically before acting.

One nurse noted*: “… sometimes they just walk in they just lift up the sheet, and I’ve said: Hold on a second here, like, let’s go outside” *(Interview, Nurse 2). Another nurse commented on her perceived role in overseeing trainees and questioning previous experience: *“You still have to watch what they are doing, and how much they know, and how confident they are… you have to ask them, like: ‘Are you good with your exams? Have you done this before?’* …” (Interview, Nurse 5).

Medical trainees acknowledged nurses’ gatekeeper role and stated that it occasionally interfered with their learning, as they felt they always needed to ask permission to see a patient or perform a skill: *“I find the nurses are very protective of their patients. So … their role is kind of the gateway to their patient … if you want to do anything with a patient you must go through them” *(Focus Group 2, Trainee 2). Nurses were also noted as patient advocates and conduits for patient preferences: *“I found that … sometimes the nurse will be, ‘Oh, like this patient won’t want to see you’”* (Focus Group 2, Trainee 3).

Nurses’ gatekeeper role was also described in the naturalistic observations:

*OBGYN residents and a nurse were discussing going to see a patient in room 2. One resident wondered whether they should get the medical student to come from triage and go with them. The nurse said that the patient had requested only women to be in the room for cultural reasons, so maybe they shouldn’t get the medical student for this patient*.

—Observation Day 4

### Engagement as a facilitator of teaching

Expressing an active interest in learning and general level of engagement were perceived as unwritten expectations and mediators of education on the BU. Nurses stated that medical trainees who were interested in learning and physically present on the unit were awarded greater teaching and learning opportunities than those who adopted more passive approaches: *“We encourage them to speak up because they’re not gonna learn anything if they sit in their room downstairs. They have to stick around …” *(Interview, Nurse 2). In some cases, a lack of engagement was perceived as disinterest: *“When people aren’t engaged and don’t want it … in those cases people will teach less … So, I think a lot of it comes off, like how engaged are they? Do they want to be here and do they want to learn?” *(Interview, Nurse 3).

Some medical trainees recognized the importance of active engagement and modified their approach to seek out learning opportunities with nurses: *“When I’m like, ‘I’m interested in gyne’, then they’re like, ‘Oh, really?’ … they’re super surprised about that and they try to get you more involved in things” *(Focus Group 1, Trainee 1). Another trainee noted that the onus is on the learner signalling interest: *“I think it’s more so you have to put yourself out there” *(Focus Group 3, Trainee 2).

That said, the level of investment in education and role modelling varied by nurse: *“And then it also depends on the nurse. So, some nurses are really, really fabulous about letting me get involved, and then other nurses not so much”* (Focus Group 2, Trainee 2). The quality and quantity of educational experience was also perceived as quite variable: *“Some of them are really experienced and willing to teach and others just are either less so or just uninterested”* (Focus Group 3, Trainee 1).

This concept is intimately related to the “gatekeeper” role on the birthing unit. Unlike some other clinical settings, nurses on the BU tend to oversee access to patients, and thus educational opportunities for medical students. Given the time sensitive nature of clinical events on the BU (i.e. inductions, deliveries, patient turnover), access is critically linked with how much one gets to learn.

### Tailoring teaching to the learner

The amount of detail nurses include in their teaching depends on experience and perceived level of knowledge of the trainee. This variability is compounded by the short period of time trainees rotate on the unit, which does not always allow for an assessment of their learning needs or goals:

*The expectations are different. The responsibilities are different, and the knowledge is different … the problem with medical students is that, you see them randomly so there is not much of a continuity there … you don’t know what their strengths … you can show them things … but it’s not … I don’t feel you give them a lot, because … the time you spend with them it might be not so much. But you still … you explain things to them*.

—Interview, Nurse 5

Given that individualized assessment of learning needs is not always feasible, and trainees tend to have teaching geared more generally to their level of medical training: “*For example, if they are a second-year medical student and they’re just coming to observe, my teaching will be a little different, it will be, maybe, a little less specific, or less intense” *(Interview, Nurse 6). This sentiment was echoed by a medical student: *“They’re not going to … expect you to do a lot or teach you a lot about that case if they think it’s like too complicated” *(Focus Group 1, Trainee 2).

This sometimes resulted in students feeling excluded when it came to involvement, procedures, and decision-making: “*They know that you’re a med student, so you can’t do too much. So, they tend to … call the residents for help, even when your name’s on the board” *(Focus Group 1, Trainee 1).

### Differences in the understanding of teaching offered by nurses in the informal curriculum

Nurses felt the scope of their teaching was very broad, and that they were able to offer specific training to medical students; students, however, did not always acknowledge the availability of this teaching. Nurses felt they had the ability to teach about *hard skills, soft skills,* and *field wisdom. Hard skills *were felt to involve procedural and administrative aspects of patient care, for example, knowing which forms to fill out, or the technical aspects of how to do a vaginal exam, how to perform fetal monitoring, etc. *Soft skills* deal more with skills such as empathic communication and bedside manners. *Field wisdom* was felt to comprise knowledge gained in the “trenches” or bedside practice which is not found in textbooks, including tacit knowledge and a sense of intuitive knowing that is rooted in context. Nurses felt that this concept related to a nurses’ ability to know how to anticipate events on the BU and to trust their “gut instincts”, often due to their many years of experience. Medical trainees acknowledged the important role of nurses’ instruction in learning *hard skills*; however, they were less cognizant of the nurses’ role in teaching *soft skills* or imparting *field wisdom*.

One nurse commented on the primacy of technical skills: “*with med students, catheterizations, IV starts, positions that help in labour, what we’re looking for, you know, progression of labour, drugs …” *(Interview, Nurse 1). Another noted that they tend to incorporate *field wisdom* in the provision of technical skill training:

*There’s technical stuff but there’s also like, experience stuff. Like things that you see throughout your years of working here, where it’s not necessarily written in stone that these things will happen, but they often happen. So, we kind of, try to let them know that*.

—Interview, Nurse 7

In addition to technical and procedural skills, the BU was considered rich with opportunities for practicing communication skills: “*Like bedside manners. You know, you talk to patient, when you talk to patient, try to talk directly to them and not look around”* (Interview, Nurse 6). These skills were regarded as essential training by the nurses: *“The main thing is though, them making sure they have a rapport with the patient before, you know, just kind of barging into the room when there’s a delivery” *(Interview, Nurse 7).

While medical trainees did not discuss the teaching of *soft skills* at length, they acknowledged the explicit expectation of rapport building with patients before attending a delivery:

*“The other expectation you hear is if you haven’t met that person, if you haven’t established a relationship, don’t even think about going in that room because we will tell you to get out of it. You have no part in that delivery”* (Focus Group 2, Trainee 2). Students did indicate, however, that they did not need to actually perform the delivery; they acknowledged that even being present provided a learning opportunity, particularly for the *hard skills* of treating patients on the BU.

## Discussion

Our study found that nurses routinely participate in the education of medical trainees; however, differing perceptions remain regarding the systematic enactment of this role. While nurses in our study seemed proud of their gatekeeper role, some medical trainees felt they missed learning opportunities because nurses either prevented their access to deliveries or did not give them a chance to introduce themselves to a patient or to enter a room. This is supported by the Canadian study by Capstick and Harley [6], which surveyed BU nurses and reported that 73% of nurses were ready to protect their patient from trainees they felt uneasy with. The same study showed that medical trainees rated their experience in OBGYN largely based on the number of deliveries they attended, and that not receiving a call to participate in the delivery of a patient they had interacted with resulted in a negative experience [6]. These opposing perceptions regarding nurses’ gatekeeper role may result in tensions in the nurse-trainee relationship, potentially making interprofessional education more challenging. Moreover, poor nurse-physician relationships could negatively influence the quality of patient care. To address this, early and ongoing interprofessional interventions during training have been proposed and shown to be effective in improving communication, clarifying professional roles, reducing conflict, and increasing collaboration [13].

Our findings of the importance of trainee engagement in facilitating learning opportunities were consistent with the aforementioned Canadian survey, which found that 64% of nurses did not make an effort to teach those who did not seem motivated, and 99% felt that an available student who demonstrated initiative would participate in more deliveries [6]. A study in the United Kingdom also found that nurses’ teaching was limited by the student’s engagement [14]. Our findings also demonstrated that nurses’ level of engagement in teaching influenced trainees’ learning; some nurses were readily available and eager to teach students, while some were not as available, regardless even of the degree of trainee engagement. To further what is described by Richards et al. [3], the concept of engagement should be considered reciprocal; that is to say that while nurses may be more eager to teach students who appear engaged, students will only be engaged if they are not denied access or activities crucial to their learning.

Medical trainees in our study reported that roles and expectations regarding their interaction with nurses during their BU experience were unclear. Similarly, Capstick and Harley [6] found that 41% of nurses believed BU nurses and medical trainees had differing perceptions about what they should expect from their rotation. Discrepancies of learning expectations have also been noted between midwives and medical trainees in an Australian study, where 6% of midwives no longer identified a role for trainees in deliveries, 17% felt trainees should support mothers with breastfeeding, and 65% agreed with trainees performing well-baby checks [15]. Thus, issues relating to teaching of the informal curriculum appear to involve more than just nurses, despite midwives and BU nurses having different roles in health care delivery.

Nurses and medical trainees in our study agreed that teaching and opportunities for participating in procedures were often tailored to the learner level. It was interesting to learn that some medical trainees recognized and accepted that medical residents or those further in training were often given priority over them to participate in procedures. However, this may not be readily evident to all trainees; as such, to address this, clear expectations of the role of medical students on rotations, in contrast to more senior trainees, should be explained at the beginning of rotations to avoid any misunderstanding or disappointment if they are not called in to participate.

Lastly, nurses in our study emphasized their role in teaching *hard skills, soft skills*, and *field wisdom*, but medical trainees did not seem to acknowledge the last two to the same degree. This is consistent with a survey of 2906 nurses who had taught medical trainees, which found that 87% of nurses felt teaching trainees was part of their job, but that their teaching contributions were not recognized by the trainees and only a few had received time allowance or financial remuneration for their teaching role [16]. Moreover, a survey of first-year medical students found that these students perceived nurses as having lower academic ability, competence, and status than doctors [17]. If nurses are not being recognized for their skills and their teaching role, this may trigger negative feelings and influence their motivation to teach, thereby affecting interprofessional education.

One possible way to address this is through the development and implementation of nurse-shadowing programs. For example, in one study [13], a half-day nurse-shadowing program for first-year medical students demonstrated that upon completion, 75% of students had more respect for nurses’ skills and knowledge, and 80% reported being more open to learning from nurses. Similar findings were reported in a Canadian study which involved interprofessional shadowing of other health care providers (in addition to nurses) by third-year medical students [18]. A systematic review also found that having a more accurate role perception improved attitudes toward collaborative practice between physician and nurses, and exposure to interprofessional teams resulted in a more positive perception of the other [8]. Moreover, another study which implemented a module in clinical skills delivered to medical students by nurses, reported that medical students acknowledged nurses’ expertise, knowledge, and resourcefulness after completing the module [19].

Our study is limited by the nature of our study design. While we attempted to gather varying perspectives from both nurses and medical trainees in relation to nurses’ teaching role, our sample was small and recruited from a single clinical setting. Further research in other clinical settings is warranted. In particular, our setting consisted of a relatively small number of students interacting with nurses on the BU. Other settings (for example, in developing countries) may have a larger number of trainees and fewer nurses, thus limiting the amount of interactions and learning that can occur. Additionally, the roles of nurses and medical students may differ depending on the clinical setting. A more hierarchical setting (with physicians as the main decision makers) may impede learning from nurses. Conversely, in other countries, nurses and midwives may do the majority of peripartum care, thus preventing educational opportunities for trainees.

Moreover, our findings may only reflect the perception of nurses and trainees interested in this topic and as such, our results may not be representative. Future lines of inquiry could also examine nurses’ perceptions of preparedness and readiness for contributing to medical education, as well as training or supports relevant to this role. Finally, educational dynamics could be explored using a sex and gender lens, though our sample did not allow for this scope of analysis.

## Conclusion

Given the valuable teaching resource nurses present, medical schools should consider acknowledging this role within the informal curriculum. While nurses in our study perceived teaching trainees as part of their role, their teaching efforts were not always recognized. This, in addition to medical trainees’ unclear expectations for their rotation, can result in tensions in the interactions between nurses and medical trainees. We recommend formalizing nurses’ teaching role to recognize their experience and expertise. This formalized teaching should occur in pre-clerkship (e.g. via job shadowing) and during clerkship. We also recommend that trainees receive clear expectations and guidelines prior to their rotation. For example, objectives and goals should be clearly outlined for, and developed with, feedback from both trainees and nurses; expected competencies to be met by the trainee should be explicitly stated; and all roles should be defined from the beginning, in order to enhance interprofessional collaboration. The results of our study may help to provide guidance for how to best pursue nurse-led trainee education.
